# Analysing NSW state policy for child obesity prevention: strategic policy versus practical action

**DOI:** 10.1186/1743-8462-4-22

**Published:** 2007-10-15

**Authors:** Lesley King, Caroline Turnour, Marilyn Wise

**Affiliations:** 1 NSW Centre for Overweight and Obesity, University of Sydney, Australia; 2 Australian Centre for Health Promotion, University of Sydney, Australia

## Abstract

**Background:**

There is increasing worldwide recognition of the need for government policies to address the recent increases in the incidence and prevalence of childhood obesity. The complexity and inter-relatedness of the determinants of obesity pose a genuine policy challenge, both scientifically and politically. This study examines the characteristics of one of the early policy responses, the NSW Government's *Prevention of Obesity in Children and Young People: NSW Government Action Plan 2003–2007 *(GAP), as a case study, assessing it in terms of its content and capacity for implementation.

**Results:**

This policy was designed as an initial set of practical actions spanning five government sectors. Most of the policy actions fitted with existing implementation systems within NSW government, and reflected an incremental approach to policy formulation and implementation.

**Conclusion:**

As a case study, the *NSW Government Action Plan *illustrates that childhood obesity policy development and implementation are at an early stage. This policy, while limited, may have built sufficient commitment and support to create momentum for more strategic policy in the future. A more sophisticated, comprehensive and strategic policy which can also be widely implemented and evaluated should now be built on this base.

## Introduction

Childhood obesity has emerged as a major public health issue only recently. To date the public discourse has focused on the magnitude of the problem and relatively narrow debates about causes and solutions. Both the public debates and initial policy responses belie the complex causal determinants of childhood obesity and the considerable policy and investment challenges facing governments.

Using the NSW Government's *Prevention of Obesity in Children and Young People: NSW Government Action Plan 2003–2007 *(GAP) [1] as a case study, this paper illustrates the argument that childhood obesity policy is still in its infancy and facing significant challenges in delivering the complex changes that will be necessary to achieve positive outcomes.

## Background

Over the past two decades there has been a significant increase in the proportion of overweight and obese children in NSW and in Australia. This is part of a worldwide trend. The most recent NSW estimate, in 2004, found that the prevalence of overweight and obesity among boys was 26% and among girls was 24% [2]. This is a significant increase from 1985 when the prevalence among boys and girls was 11–12% [2]. The increases in the proportion of children who are overweight or obese have profound social, cultural, emotional, medical and economic consequences. For these reasons, childhood obesity prevention has become a topic of community concern and media debate, as well as policy development.

### Public policy

The term 'policy' refers to a procedure or guide to action to achieve intended goals and, purportedly or actually, functions to set priorities and guide resource allocation. While policy can take the form of a formal statement, that is explicit and open to comment and accountability, this is not a necessary aspect, and policy can remain unwritten [3]. 'Public policy' refers to policy at any level of government [4].

It is widely understood that the policymaking process is influenced by social factors including cultural norms, stakeholder interests, power relations, ideological beliefs and organizational cultures, as well as scientific knowledge [5].

Policy implementation has been less extensively researched. Stated simply, it involves putting proposed actions into effect. However, policies vary in the ease with which they can be implemented; and jurisdictions vary in their capacity to implement new policies. Disjunctions between policy and implementation are commonplace. Furthermore, differences in what politicians and the public expect, and what the infrastructure can deliver, are common in public health [6].

### Childhood obesity prevention policy

In Australia the first policy response to the emerging problem of obesity came from the National Health and Medical Research Council (NH&MRC), which in 1997 produced the report *Acting on Australia's weight: a strategic plan for the prevention of overweight and obesity *[7]. The report focused on promoting physical activity and healthy diet in key settings, acknowledged that increased prevalence of overweight and obesity in the population was due to lifestyle and environmental factors, and singled out children and adolescents as a target group. Despite the report's strengths, its recommendations were largely ignored at that time [8].

Five years later, in 2002, the NSW Government held the NSW Childhood Obesity Summit in the NSW Parliament [9], putting childhood obesity on the political and public agenda, at least at that time. While the precise circumstances and impetus which led to the government convening this summit have not been documented, the interplay of social factors and competing interests at the Summit has been reported elsewhere [8]. The NSW Summit did provide recognition that childhood obesity was an emerging population health problem and that there were multiple stakeholders involved in creating the problem and, potentially, in addressing it.

The 2002 Summit marked the beginning of a planned, formal and serious response to childhood obesity in NSW and Australia. Action in NSW was followed by the formulation of a national approach by the National Obesity Taskforce [10] and policies in other states and territories [11]. It also coincided with the development of policies internationally, such as in Europe and North America (for example, *Preventing Childhood Obesity: Health in the Balance *[12]).

### Childhood obesity prevention policy challenges

Child obesity policies have had to deal with three fundamental issues:

▪ The complexity of causal determinants which imply that a broad range of potential interventions will be required.

▪ The lack of a well-developed body of evidence on the effectiveness of interventions.

▪ The fact that many of the necessary responses are outside the direct ambit or control of the health sector.

Complex causal explanations of population health problems pose genuine policy challenges [13], and this is the case for the prevention of childhood obesity. Debates about the relative contributions of different determinants, as well as the difficulties of proving that interventions are effective, add to the contestability of policy options. For example, the food industry rejects calls for reforms in that sector by shifting the blame for childhood obesity onto the lack of exercise [14].

Most of the multiple determinants are the responsibility of sectors other than health, so that many non-health sectors must be engaged in formulating and implementing the policy responses that will be necessary for effective solutions [15]. In recognition of the complexity of childhood obesity prevention, the International Obesity Task Force (IOTF) has emphasised the importance of a comprehensive public health approach and developed a list of principles and recommendations to guide policy formulation [9].

While it is expected by the population health sector that policy be based on research-derived evidence of effectiveness, this is not always the case or even possible. The complexity of determinants and responses makes the accumulation of evidence about effective interventions particularly difficult [16,17]. In some cases, evidence points to actions that are politically unacceptable, and evidence is more passionately contested. The contested nature of policy means that policy analysis studies often focus on the dynamics of each step in policymaking.

Alternatively, the focus of policy analysis can be on features of the policy itself, such as:

▪ Was it the right policy? For example, this form of analysis may take account of whether policy was evidence-based, and whether it was appropriate to the target group and social context.

▪ Was the scope and scale adequate? Was the scale and intensity of effort sufficient to achieve the policy goals?

▪ Was the policy effective? Did the policy achieve stated or intended goals or performance indicators? Were there any unintended consequences?

▪ Was it feasible? That is, did the available resources and infrastructure enable the policy to be implemented?

These questions were considered in this study on the implementation of childhood obesity prevention policy in New South Wales.

### Preventing obesity in children and young people: the NSW Government Action Plan 2003–2007 (GAP)

In response to the 2002 Summit, childhood obesity prevention became a NSW Government priority, and the NSW Government launched the *Prevention of Obesity in Children and Young People: NSW Government Action Plan 2003–2007 *(GAP) [1]. This plan represented initial policy steps recommended by participants at the Summit (a combination of population health and clinical experts, industry/sectoral representatives, community representatives and politicians) to address the social, economic, environmental and behavioural factors contributing to the problem of childhood obesity.

The GAP identified 34 actions that NSW Health Department, NSW Department of Education and Training, NSW Department of Community Services, NSW Department of Sport and Recreation and NSW Roads and Traffic Authority agreed to implement in order to expand their contributions to the prevention of obesity in children and young people. The priority areas comprised:

▪ Healthy Schools

▪ An Active Community

▪ Supporting Parents

▪ Healthy Child and Out-of-School Care

▪ Community Understanding

▪ Increasing Our Knowledge

▪ Governments and Industry and the Community Working Together.

## Methods

This paper describes a systematic analysis of the implementation of the NSW GAP policy, with specific attention being given to the content, scope and feasibility for implementation. The analysis did not cover the policy development process, or the effectiveness of the policy in achieving specific outcomes, as information about these was not available at the time of the study.

Policy guides published by the IOTF and World Health Organization (WHO), and written by international experts, provided appropriate and internationally authoritative benchmarks against which to assess the quality and implementation of the GAP. The IOTF's principles and recommended actions outline the key features of a public health approach to obesity prevention [16] and provide reference points against which to assess scope and content of the actions proposed in the GAP. The WHO Stepwise Framework for Chronic Disease Prevention Policy implementation steps (see Table 1) provides criteria against which to assess the scope and scale of policy implementation [18,19]. It distinguishes core actions that can be implemented with existing resources and structures, from those that require an expanded level of resources and those that would require substantial new resources. Its application to the GAP explicitly links the availability of resources with the different stages of policy implementation.

**Table 1:** WHO Stepwise Framework for Chronic Disease prevention – policy implementation steps

**Policy implementation steps**	**Description**
Implementation Step 1 **CORE**	Interventions that are feasible to implement with existing resources in the short term
Implementation Step 2 **EXPANDED**	Interventions that are possible to implement with a realistically projected increase in, or reallocation of, resources in the medium term.
Implementation Step 3 **DESIRABLE**	Evidence-based interventions which are beyond the reach of existing resources.

(World Health Organization 2005)

Based on benchmarks derived from the documents noted above, the authors defined criteria to guide the policy analysis. The benchmarks were used in order to minimise arbitrary judgements, and provide legitimacy and credibility, to ensure that the evaluation was acceptable and useful to policymakers.

The assessment of the GAP was conducted by one of the authors (CT), who reviewed documentation and interviewed stakeholders in each of the agencies responsible for policy implementation. The findings were checked by another author (LK) and reviewed by the interviewees. All authors were involved in discussion and interpretation of the findings to ensure the rigour and credibility of results.

## Results

### Scope and potential to prevent childhood obesity

Table 2 presents a summary of how the actions correspond to the IOTF principles and recommendations. Overall, the proposed actions displayed features that correspond to IOTF recommendations and principles. A number of relevant government departments were involved, actions were taken and program managers did respond to the issue and link their actions to the prevention of childhood obesity. However, the policy fell short of the IOTF recommendations, in terms of breadth, sustainability of changes, and scale of actions to be implemented.

**Table 2:** Analysis of GAP actions according to IOTF recommended actions and principles

**IOTF Recommended Action (1 to 5)**	**Comments**
**1. **Address both dietary habits and physical activity patterns of the population.	Overall, actions cover both physical activity and nutrition, but tend to be identified and implemented separately, in different settings and jurisdictions and different target groups. Information-dissemination actions were the only actions that addressed physical activity and food consumption in an integrated way.
**2. **Address both societal and individual level factors.	The *GAP *contains a mix, with the majority of actions directed at social and environmental factors in specific settings (e.g. school canteens).
**3. **Address both immediate and distant causes	Focus is on behaviours in everyday settings, rather than social, cultural factors
**4.** Address multiple focal points and levels of intervention (i.e. national, regional, community and individual levels)	While there is a mix of local and state level action, in many cases the local projects are limited to a small number of sites and unlikely to achieve widespread reach or population effect, unless they were implemented on a major scale across the state, or there is a clear process for staged dissemination and statewide implementation.
**5.** Build links between sectors that may be otherwise viewed as independent.	The *Action Plan *brings together programs across key portfolios and creates a significant cross-sectoral agenda and basis for collaboration. However, there are a number of factors that are not addressed, including transport, safety, media and food supply.
**6.** Include both policies and programmes.	The *GAP *is weighted more towards program implementation, rather than policy development. Only three actions relate to changing policy. However, some of the programs have a basis in existing policy, such as earlier commitments to build off-road cycleways.

**IOTF PRINCIPLES (1 to 10)**	

**Principle 1. **Education alone is not sufficient to change weight-related behaviours. Environmental and social interventions are also required to promote and support behavioural change.	While the majority of actions include environmental interventions, many are small scale and local.
**Principle 2. **Action must be taken to integrate physical activity into daily life, not just to increase leisure time exercise.	Seven actions within the *Plan *fit this principle.
**Principle 3. **Sustainability of programmes is crucial to enable positive change in diet, activity and obesity levels over time.	The *GAP *is primarily concerned with initiating discrete actions, and does not emphasise structural changes or sustainability.
**Principle 4. **Political support, intersectoral collaboration and community participation are essential for success.	*GAP *was initiated with the significant political support and community participation associated with the 2002 Childhood Obesity Summit
**Principle 5. **Acting locally, even in national initiatives, allows programmes to be tailored to meet real needs, expectations and opportunities.	Many of the *GAP*'s actions support local initiatives. While this approach can support appropriate and tailored approaches, it is important to note that many areas and communities will not be recipients of local initiatives, as actions have only been implemented in selected locations and not universally across NSW
**Principle 6. **All parts of the community must be reached – not just the motivated healthy.	Many *GAP *actions adhere to this principle through adopting a targeted or local approach. However, as this occurred in the context of small projects, they did not reach wide sections of the population.
**Principle 7. **Programmes must be adequately resourced.	While NSW Treasury did not directly allocate additional resources, NSW Health allocated additional funding, totalling $12 million over three years (2004/5 to 2006/7). These funds were concentrated on two new initiatives, the Healthy Schools Canteen Strategy and the establishment of the Centre for Overweight and Obesity. The recent investment of $7.5 million over 5 years by NSW Health and Hunter New England Area Health Service for a large Area-wide, intensive demonstration project (Hunter New England child obesity prevention program), represents a major investment by NSW Government.
**Principle 8. **Where appropriate, programmes should be integrated into existing initiatives.	This principle is central to the *GAP*'s design, as 50% of actions were identified as core actions for existing agencies.
**Principle 9. **Programmes should build on existing theory and evidence.	*GAP*'s actions are based on best available evidence, as well as health promotion theory. Note that there was limited evidence regarding effective interventions.
**Principle 10. **Programmes should be properly monitored, evaluated and documented. This is important for dissemination and transfer of experiences.	The *GAP *did not specify any impact (e.g. children's eating and physical activity behaviours) or outcome (e.g. population weight status) indicators for reporting and monitoring purposes. The complexity of multiple determinants influencing children's weight status makes it difficult to measure and attribute the direct impact of the actions listed in the *Plan*. This is particularly true for analysing the contribution of actions that have obesity as a secondary objective.

GAP did reflect a collaborative approach between government agencies beyond the health sector, but did not extend to include all relevant sectors, such as urban or transport planning, or agriculture. The focus of the policy was on programs and specific, existing initiatives, rather than an overall strategic approach. Most of the actions that had been taken were not sustainable. Half of the 34 proposed actions fitted within agencies' core business, while others involved an extension of existing roles (Table 3). Importantly, GAP did not define specific outcomes or set evaluation indicators, so that there was no precise way to measure its effectiveness.

**Table 3:** Classification of GAP proposed actions as core and expanded

**Core implementation actions** (Interventions that are feasible to implement with existing resources in the short term)	**Expanded Implementation Actions** (Interventions that are possible to implement with a realistically projected increase in, or reallocation of, resources in the medium term)
**HEALTHIER SCHOOLS**	
School Sport Foundation	Healthy School Canteens Strategy – policy
Revitalization of secondary school sports program	Healthy School Canteens Strategy – information
Support materials for teachers to implement and supervise sport programs	NSW Health will increase funding for NSW School Canteen Association
Professional support for teachers to implement new Years 7–10 Personal Development, Health and Physical Education syllabus	
Distribute resources about school based strategies for getting students active	
Rock Eisteddford Challenge and Croc Rock Festivals	
**AN ACTIVE COMMUNITY**	
Modify the Active Communities Grants Scheme to increase the focus on preventing childhood obesity	Funding for Public Health Policy Officer position in Local Government Association
Work with members of the Active Communities Network to strengthen the understanding of childhood obesity issues	
The promotion of walking and cycling through community based initiatives like Bike Week.	
Building off-road cycleways	
Support local government to develop and construct local cycleway networks.	
Provide financial assistance and expertise for local government to develop Pedestrian and Access Mobility Plans.	
**SUPPORTING PARENTS**	
The Early Intervention Program and Flexible Child Care and Family Service projects	Additional funding to NSW Branch of the Australian Breastfeeding Association
Support and information to parents about healthy weight through Families First (early intervention, home visiting program)	Development and dissemination of NSW Breastfeeding policies
	Expert Taskforce on O&O support and treatment services
**HEALTHY CHILD AND OUT-OF-SCHOOL CARE**	
Nutrition information and advice on good practice in physical activity for children services and out-of-school hours programs	Active Out-of-School Hours Care pilot programs: ◦ Trialling of various ways of providing physical activity ◦ Development and trialling of a training program for OSHC staff ◦ Development and trialling of a start-up package for Centres ◦ Trialling of physical activity policy guidelines for OSHC centres ◦ Evaluation of the key elements of success.
Nutrition and physical education training programs for child care professionals	Develop and trial a physical activity training package for staff working in out-of-school hours care (OSHC) centres; based on the competencies identified in the Certificate IV 'Train the Trainer' Physical Activity for Children and Youth.
**COMMUNITY UNDERSTANDING**	
NSW Health will develop and maintain the overweight and obesity website	State-wide community education campaign
	Publish an easy to use compendium of nutrition and physical activity related information and resources on NSW Health's Childhood Obesity Website.
	Develop a user-friendly, online training program providing information on physical fitness, nutrition and healthy lifestyle options for children
**INCREASING OUR KNOWLEDGE**	
	Establishment of Centre for Overweight and Obesity and Australian Child and Adolescent Obesity Research Network (ACAORN) to support research.
	Schools Physical Activity and Nutrition Survey
**GOVERNMENTS, INDUSTRY AND COMMUNITY WORKING TOGETHER**	
	Not specified – Government, Industry and Community Working Together

## Discussion

The GAP's array of seven priority topic areas, five responsible agencies and 34 actions represented cross-sector commitment to action. The identified actions were clearly designed to be implemented within the constraints of existing resources in the short-term, or with small increases in resources in the medium term. However, the small scale on which some of the actions were implemented (for example, community based projects in only a small number of communities) suggests that the likelihood of achieving any population level changes in behaviour, environment or weight status was low. On the other hand, such interventions may contribute to evidence of efficacy that may, in turn, be useful in arguing for greater investment and a more sustained, large-scale response in the future.

It can be argued that this modest policy, with a mix of core and expanded actions, was appropriate in the early years of a government-led response to a major population health problem for which there is limited evidence upon which to base investment in major new interventions. In 2002, when the NSW Childhood Obesity Summit was held, there was limited evidence in relation to effective interventions to address childhood obesity, and to guide decisions about interventions with the greatest potential to produce health benefits. The practical approach that was adopted by GAP can be perceived as staking out a place for obesity prevention on the public policy agenda and harnessing existing infrastructure.

However, the focus on a narrow set of practical actions linked with existing core business, rather than strategic policy, remains open to critique. The GAP presented a list of actions that were based on Summit recommendations, which the relevant government departments agreed to complete by 2007. The Summit resolutions themselves have been critiqued as being potentially diluted and narrow because they emerged from negotiations between competing stakeholders, including food industry groups [8].

The GAP was not a strategic response, in the sense of providing a long-term vision, specific goals or a broadly integrative or sustainable approach, although this is what the IOTF has recommended. GAP provided a starting point for action, rather than a fully developed policy that tackled fundamental challenges related to the complexity of causes, building large scale cross-sector initiatives or testing new interventions. While a comprehensive response that involved community, government and private industry sectors was required to tackle childhood obesity, GAP concentrated on practical activities in a limited number of government sectors. The activities can be seen as avoiding controversial political manoeuvres, and, as often occurs in health promotion, only minimally responding to the policy challenges [20]. As Lewis [21] recognises, policy solutions that are deemed acceptable and feasible or that have gone through 'a softening up process', may be preferred, as they avoid confrontation and have greater likelihood of being implemented.

As with program evaluation, policy analysis ideally draws upon a mix of information sources and analytic tools to examine the content, implementation process and outcomes. The use of explicit criteria in this analysis, to assess the breadth and infrastructure for obesity prevention policy, proved to be an acceptable and constructive approach to this study. The tools gave direction and authority to the process of identifying strengths and weaknesses in the content and implementation of GAP. However, these tools could not provide a basis for assessing the achievement of outputs or outcomes. The generic and flexible nature of each of the analytic frameworks also meant that they did not provide a basis for checking if the implemented initiatives actually produced outcomes. Even if the policy could be shown to have met the criteria more completely, this would not itself guarantee the achievement of specific outcomes.

## Conclusion

This case study revealed that the implementation of the GAP contributed to the development of infrastructure and resources for childhood obesity prevention. In addition, the process of implementing GAP has lead to significant cross sector collaboration, which is important, as many of the causal determinants of obesity lie outside the health sector. Flagship actions, such as the Healthy Schools Canteen Strategy, also provided a valuable symbol for the cultural and behavioural changes needed as part of the long-term process of slowing current trends in childhood obesity. Nevertheless, it is important not to under-estimate the range and extent of further changes and resources that will be required to reverse increasing rates of childhood obesity.

This analysis can be used to guide the next stage of public policy. Childhood obesity prevention continues to form an important public health goal, and has successfully captured media and political attention in Australia and spurred governments to hold summits and develop policies. It is critical that attention is now directed to formulating more comprehensive responses, which tackle the issue on a scale appropriate to the problem. A more strategic response needs to include a wider range of government sectors and recognise the links between health, obesity and economic levers, social infrastructure, and human capital and productivity. Policy leadership will be critical to extending the GAP activities evaluated in this case study, into a more integrated and strategic policy approach in the future.

## Competing interests

The NSW Centre for Overweight and Obesity was funded as part of the *Prevention of Obesity in Children and Young People: NSW Government Action Plan 2003–2007*, which is the subject of the research reported in the article.

The funding body was not involved in the collection, analysis or interpretation of data.
